# Supplementary material for: Effects of Feeding and Drinking Behavior on Performance and Carcass Traits in Beef Cattle
Source: Animals (Basel). 2022 Nov 18;12(22):3196. doi: 10.3390/ani12223196 (PMC9686570; doi:10.3390/ani12223196)
Supplement: Supplementary file 1 [file animals-12-03196-s001.zip › animals-2024325-supplementary.pdf]

Table S1. Percentage of ingredients and nutrient composition of the diet provided to animals during the feed efficiency test and formulated for 1,200 kg/d.

**Ingredients (% DM)**	
Sorghum silage	60.0
Soybean meal	13.0
Ground corn	25.0
Mineral salt[1]	1.75
Urea	0.25
Forage to concentrate ratio	60:40
**Nutrients**	
Dry matter, %	52.4
Crude protein, % DM	11.2
Ash, % DM	4.63
Ether extract, % DM	2.13
Neutral detergent fiber, % DM	40.6
Acid detergent fiber, % DM	24.4
Gross energy, Mcal/kg	3.77
Non-fiber carbohydrates, DM %	41.5
Total digestible nutrients[2], DM %	70.2

DM: dry matter. [1]Composition: 16 g/d phosphorus, 34 g/d calcium, 13 g/d sodium, 4.4 g/d sulfur, 1.6 g/d magnesium, 720 mg/d zinc, 200 mg/d copper, 140 mg/d manganese, 16 mg/d cobalt, 16 mg/d iodine, and 3.6 mg/d selenium. [2]Values calculated using the equation of Weiss (1999) [74].

Table S2. Descriptive statistics (mean, standard error, minimum and maximum) of the efficiency, carcass and behavior traits observed in Caracu cattle.

Trait	Mean	SE	Min	Max
BWi (kg)	218.75	4.21	119.00	303.00
BWf (kg)	296.75	4.16	187.00	414.00
ADG (kg/d)	1.01	0.02	0.42	1.46
DMI (kg/d)	8.48	0.09	6.06	10.35
$BW^{0.75}$ (kg)	66.38	0.81	45.56	81.44
WI (L/d)	20.79	0.40	11.65	29.92
REA (cm^2)	57.00	1.01	36.00	80.70
BF (mm)	2.46	0.14	0.00	6.80
RF (mm)	4.56	0.11	2.30	8.36
NW (number/d)	2.87	0.03	2.08	3.44
FVW (visits/d)	5.42	0.10	3.00	7.86
DD (min/d)	69.80	3.66	12.00	171.00
DR (L/min)	1.00	0.31	0.23	2.72
NF (number/d)	10.22	0.11	7.15	11.90
FVF (visits/d)	58.51	1.61	23.93	105.57
FD (min/d)	160.14	2.06	100.77	209.13
FR (kg/min^1)	0.09	0.001	0.04	0.19
RWI - low	2.08	1.84	0.07	8.01
RWI - high	-2.52	1.46	-7.45	-0.09
RFI – low	0.36	0.28	0.02	1.24
RFI - high	-0.43	0.27	-1.15	-0.04

BWi: body weigh initial; BWf: body weigh final; ADG: average daily weight; DMI: dry matter intake; $BW^{0.75}$: mid-test metabolic weight; water intake (WI); REA: rib eye area; BF: backfat thickness; RF: rump fat thickness; NW: number of water troughs visited per day; FVW: frequency of visits to the water through with intake; DD: drinking duration; DR: drinking rate; NF: number of feed bunks visited per day, FVF: frequency of visits to the feed bunk with intake; FD: feeding duration; FR: feeding rate.

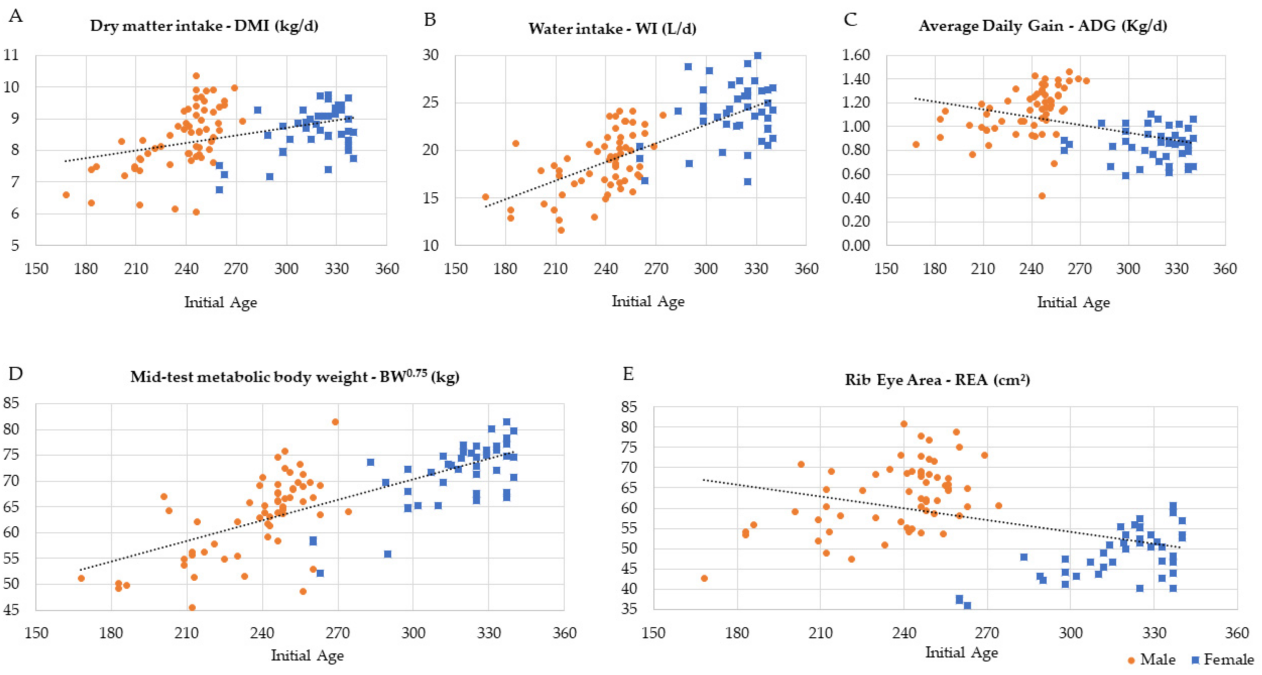

Figure S1. Distribution of the traits according to the initial age of Caracu males (orange circles) and females (blue squares). A. Dry matter intake (DMI), B. Water intake (WI), C. Average daily gain (ADG), D. Mid-test metabolic body weight ($BW^{0.75}$), and E. Rib eye area (REA).

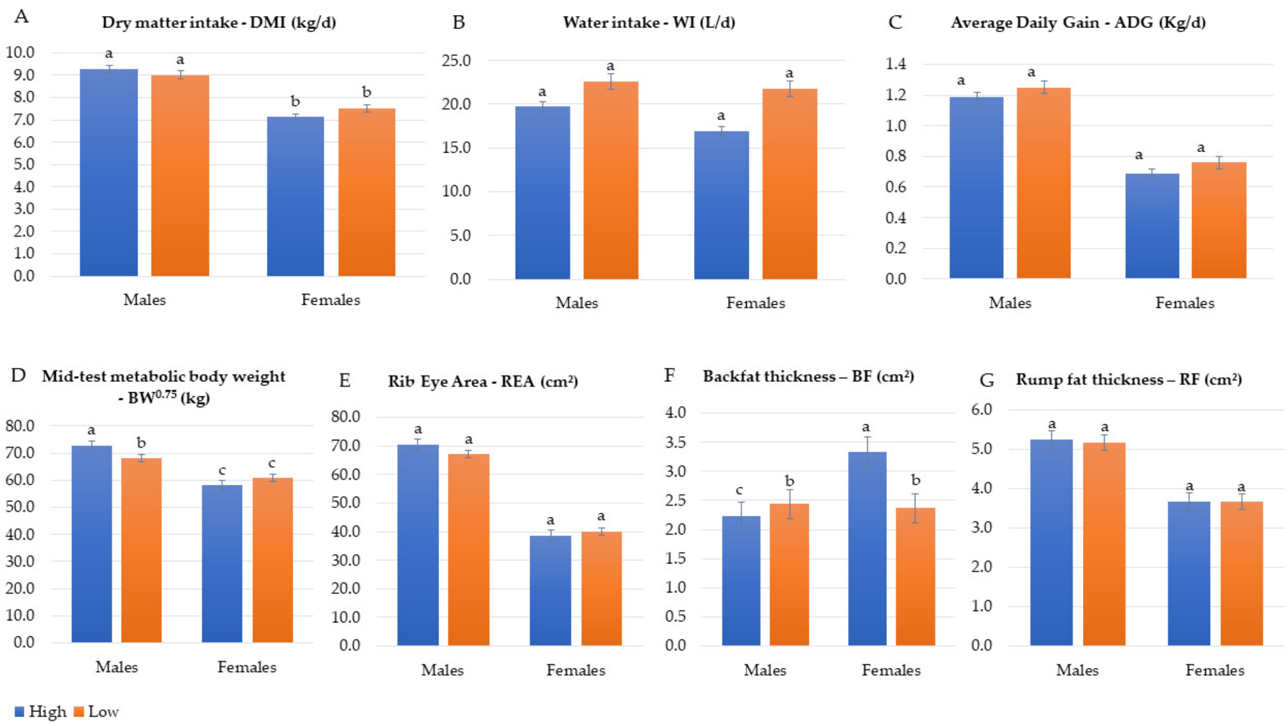

Figure S2. Interaction between sex and RWI class for dry matter intake (A), water intake (B), average daily gain (C), mid-test metabolic body weight (D), rib eye area (E), backfat thickness (F), and rump fat thickness (G).

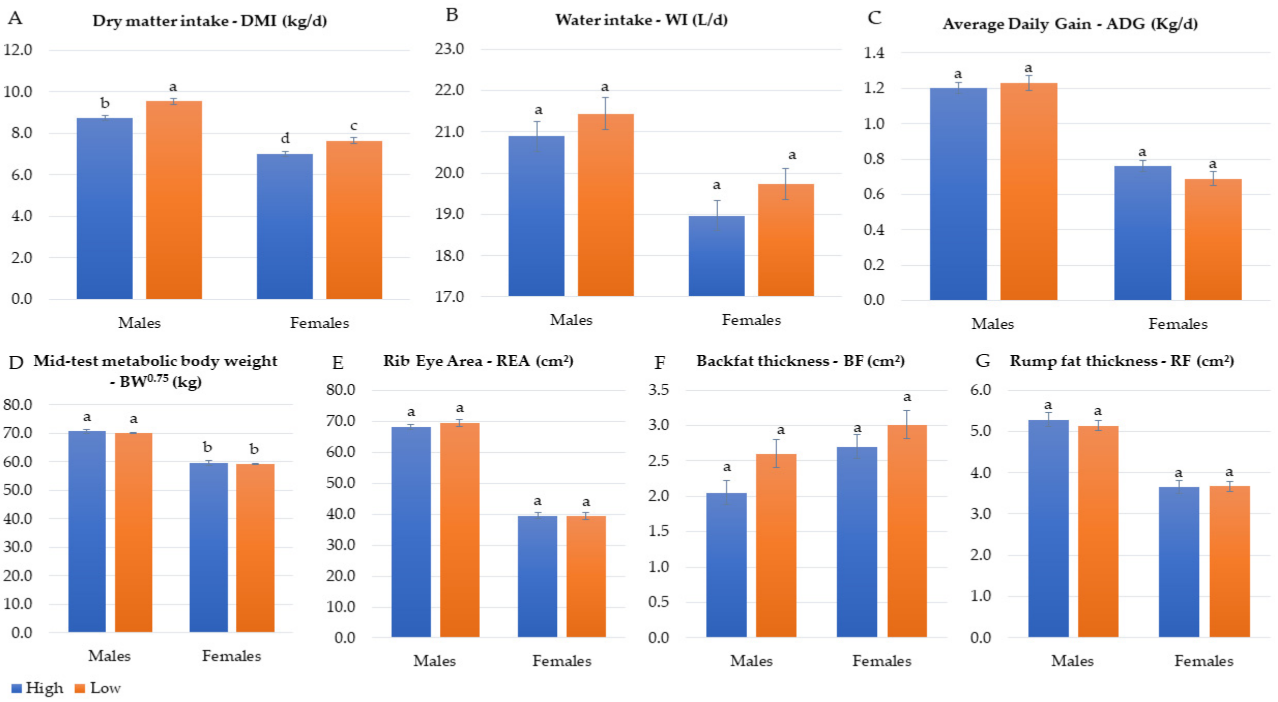

Figure S3. Interaction between sex and RFI class for dry matter intake (A), water intake (B), average daily gain (C), mid-test metabolic body weight (D), rib eye area (E), backfat thickness (F), and rump fat thickness (G).

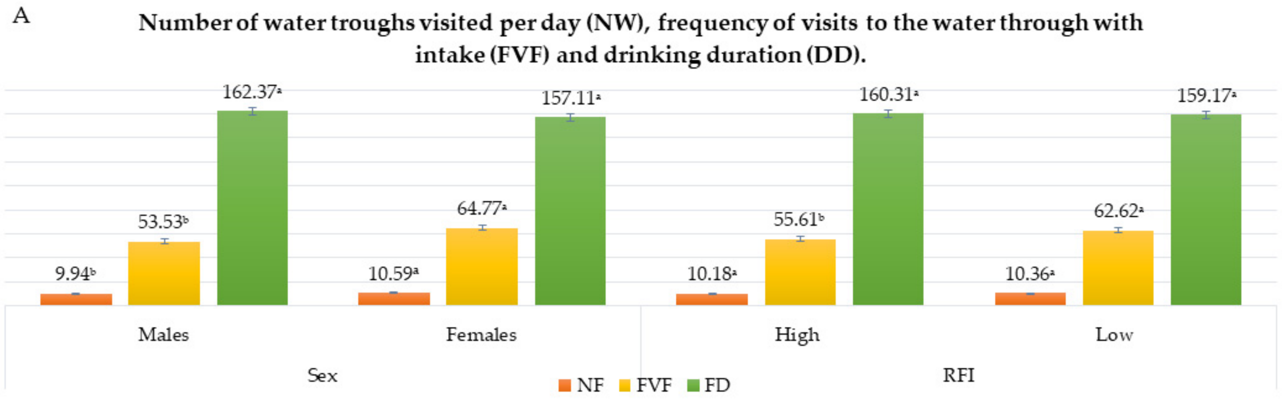

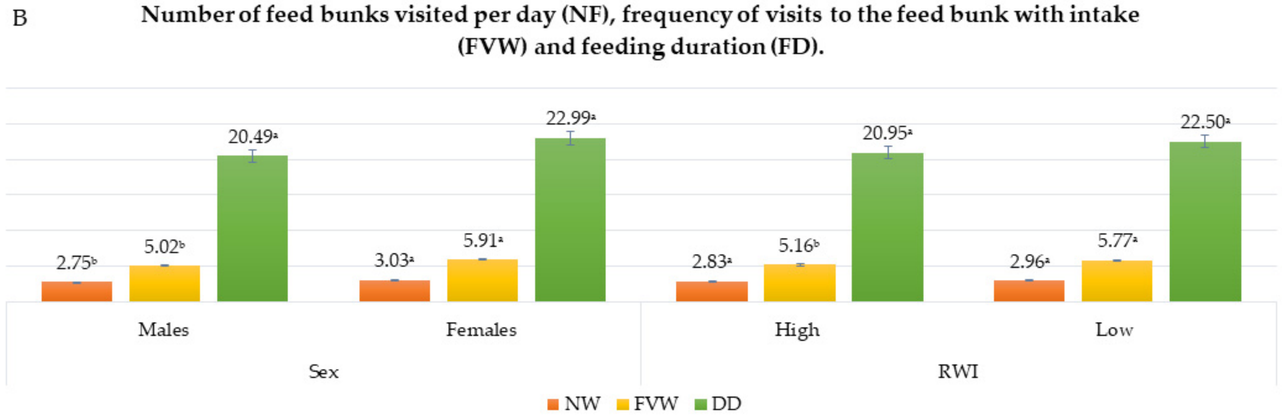

Figure S4. A. Behavior traits at the feed bunks (NF, FVF, and FD) according to sex and RFI class. B. Behavior traits at the water troughs (NW, FVW, and DD) according to sex and RWI class.

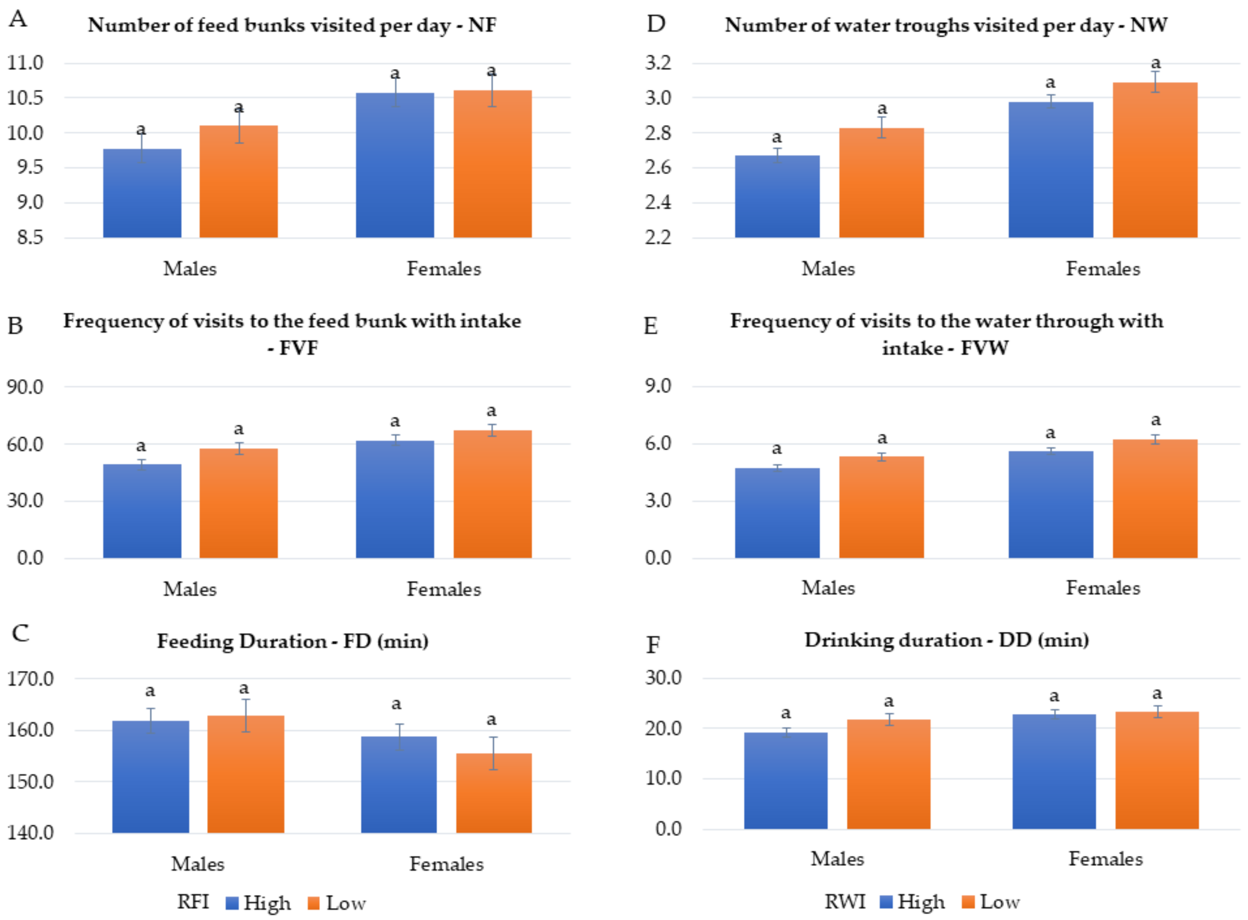

Figure S5. Behavior traits at the feed bunks (A: NF, B: FVF, and C: FD) according to interaction between sex and RFI class. Behavior traits at the water troughs (D: NW, E: FVW, and F: DD) according to interaction between sex and RWI class.
